# Macrophages suppress CD8 + T cell cytotoxic function in triple negative breast cancer via VISTA

**DOI:** 10.1038/s41416-025-03013-5

**Published:** 2025-05-02

**Authors:** Maidinaimu Abudula, Yuliana Astuti, Meirion Raymant, Vijay Sharma, Michael C. Schmid, Ainhoa Mielgo

**Affiliations:** 1 Department of Molecular and Clinical Cancer Medicine, University of Liverpool, Liverpool, UK; 2 School of Medicine and Department of Molecular and Clinical Cancer Medicine, University of Liverpool, Liverpool, UK; 3 Department of Cellular Pathology, Liverpool Clinical Laboratories, Liverpool University Hospitals NHS Foundation Trust, Liverpool, UK

**Keywords:** Cancer microenvironment, Breast cancer

## Abstract

**Background:**

Immunotherapy targeting negative immune checkpoint regulators to enhance the anti-tumour immune response holds promise in the treatment of TNBC. V-domain Ig suppressor of T-cell activation (VISTA) is an immune checkpoint molecule, known to be upregulated and involved in modulating tumour immunity in TNBC. However, how VISTA affects immune response and its therapeutic potential in TNBC remains unclear.

**Method:**

Here, we examined VISTA expression and cellular distribution in TNBC patients’ samples and pre-clinical TNBC mouse model. Functional assays were performed to assess the impact of VISTA blockade on macrophage phenotypes, CD8 + T cell infiltration and activation, and overall anti-tumour immune response.

**Results:**

In this study we show that VISTA expression levels are increased in TNBC patients’ samples and pre-clinical mouse models compared to non-involved breast tissue and VISTA is mainly expressed on tumour infiltrating macrophages and neutrophils. Blocking VISTA reverts macrophages immunosuppressive phenotypes, increases CD8 + T cell infiltration and activation, and enhances an anti-tumour immune response. Mechanistically, we show that neutralising VISTA on macrophages enhances their immune-stimulatory functions and inhibits the suppressive effect of macrophages on CD8 + T cells activation.

**Conclusion:**

These findings provide the rationale for the development of anti-VISTA targeting strategies in the treatment of TNBC.

## Background

Triple negative breast cancer (TNBC) is a highly heterogenous and aggressive subtype of breast cancer (BC), constituting 15–20% of all BC cases, and notably lacks effective targeted treatments [1]. Conventional chemotherapy and radiotherapy are the frontline therapy for patients with TNBC. However, patients often develop drug resistance, fail to achieve pathological complete response, and have shorter survival rate compared to other BC subtypes [2].

TNBC is recognised for its high immunogenicity, characterised by increased presence of tumour-infiltrating immune cells and higher tumour mutational burden, which renders the disease a promising candidate for immunotherapy [3]. Immune checkpoint inhibitors (ICIs) targeting inhibitory immune checkpoint molecules have shown therapeutic efficacy across various malignancies over the last decade [4]. Specifically, ICIs targeting programmed cell death-1 (PD-1) and PD-1 ligand 1 (PD-L1) have been approved for treating patients with locally advanced or metastatic TNBC [5]. However, only approximately 5% of patients exhibit durable response to Atezolizumab (anti-PD-L1 inhibitor). Although combining Atezulizumab with chemotherapy has increased the response rates to around 10%, the majority of patients still do not respond adequately to the combination treatment [6]. This suggests the involvement of additional inhibitory pathways that tumour cells may exploit to evade immune destruction. Therefore, understanding the expression and function of other inhibitory checkpoint molecules present in TNBC holds promise for developing more effective therapeutic regimens.

V-domain Ig suppressor of T-cell activation (VISTA, gene:*VSIR*), also known as PD-1H, is an inhibitory immune checkpoint molecule of the B7 protein family [7, 8]. VISTA exhibits high expression on myeloid cells and intermediate to low levels of expression on T cells [9]. Previous studies have shown that VISTA plays a crucial role in regulating both auto-immune and anti-tumour immune responses, and exerts its function as both a ligand and a receptor [9–11]. While other immune checkpoint molecules primarily exert immunosuppressive effects by regulating T cell activation, VISTA exhibits a multifunctional role in regulating the function of both lymphoid and myeloid cells within the TME [12]. The expression of VISTA in the tumour has been associated with poor survival in patients with bladder cancer, glioma and melanoma [13–15]. Furthermore, VISTA expression can be further upregulated upon ICIs therapy in melanoma and prostate cancer, potentially contributing to adaptive immune resistance [16, 17]. In mouse models of melanoma and bladder cancer, genetic deletion of *Vsir* or administration of anti-VISTA blocking antibodies has been shown to enhance T cell-mediated anti-tumour immune response and inhibit tumour growth [18–20]. This effect has been attributed to the role of VISTA in reducing myeloid cell infiltration and increasing T cell infiltration in the TME [19]. Targeting VISTA on antigen presenting cells (APCs) within the TME attenuates its suppressive effects on CD8 T cell activation while increasing antigen presentation and pro-inflammatory functions of APCs in colon cancer [21, 22]. Collectively, these observations suggest that VISTA is a crucial immune checkpoint regulator that regulates both CD8 + T cell and myeloid cell functions in tumours.

VISTA has emerged as a significant target for investigation in TNBC, as evidenced by its upregulation in human TNBC tumour tissue in comparison to other immune checkpoints [23]. Notably, VISTA expression in TNBC tissue is nearly twofold higher than in adjacent breast tissue [23]. However, the mechanism by which VISTA regulates the anti-tumour immune response and the therapeutic benefit of targeting VISTA in TNBC remains largely unexplored. In this study, we show that VISTA expression is upregulated on TNBC tissue, and predominantly expressed by macrophages and neutrophils. Pharmacological blocking of VISTA promotes the repolarization of macrophages towards a pro-inflammatory phenotype, increases CD8 + T cell infiltration and activation within the TME, thereby augmenting anti-tumour immunity in TNBC. These findings underscore the potential therapeutic implications of targeting VISTA in TNBC.

## Method

### Cell line

The murine breast cancer cells Py230 (estrogen and progesterone-receptor negative and HER2 low) were generated in Ellies lab (University of California, San Diego) and obtained from spontaneous mammary tumours arising in MMTV-PyMT of a C57BL/6 female mice background by serial trypsinization and limited dilution [24]. Py230 cells were cultured in DMEM/F-12 (Gibco) culture media supplemented with 10% FBS and 0.5% MITO serum extender (Corning #355006), antibiotic antimycotic solution (10 U/mL penicillin, 0.1 mg/mL streptomycin, and 0.25 ug/mL amphotericin B) (Sigma) in an incubator with humidified air and 5% CO2, at 37 °C. Cells were regularly tested negative for *mycoplasma*.

### Mice

All mice used in this study were 6–8 weeks old female C57BL/6 mice and obtained from Charles River Laboratories. Mice were kept under specific pathogen-free conditions at the Biomedical Service Unit at the University of Liverpool. All animal experiments were conducted in accordance with the current UK Home Office regulations and under an approved project license P16F36770.

### Syngeneic orthotopic breast cancer model

In the orthotopic syngeneic breast cancer model, 2 × 10^6^ Py230 cells were injected into the mammary fatpad of C57BL/6 6–8 weeks old female mice. Established tumours were measured with calipers two-three times a week and treatment started when the tumours measured between 1.0 and 1.3 cm mean diameter. For anti-VISTA mAb (13F3, BioXCell) treatment, mice were administered intraperitoneally (i.p.) with either hamster control IgG antibody or anti-VISTA mAb (300 µg per mouse). Following treatment strategies, mice were euthanised by Schedule 1 method of cervical dislocation after 24 h and tissue harvested for downstream analysis.

### Analysis of tumour-infiltrating immune cells

For primary tumours, single-cell suspensions were generated from mechanical and enzymatic digestion with Collagenase P (1 mg/ml; Roche) in Hank’s Balanced Salt Solution (Gibco, 24020091) at 37 °C for 30–40 min as previously described [25]. Cells were incubated with 0.05% trypsin (Gibco) at 37 °C for 5 min, then filtered through 70-μm cell strainer to remove the debris and proceed to remove the red blood cells using RBC Lysis Buffer (BioLegend). For lung tissue, single-cells were prepared from mechanical and enzymatic digestion with Collagenase D (2 mg/ml; Roche) and DNaseI (0.5 mg/ml; brand) in Hank’s Balanced Salt Solution, and processed as described above.

### Flow cytometry

Primary tumours and lung single-cell suspension were resuspended in MACS buffer (0.5% BSA, 2 mM EDTA in PBS), and incubated on ice with CD16/CD32 (Mouse BD Fc Block, BD Biosciences, 553142) for 10 min. For cell surface staining, cells were stained with SYTOX Blue viability marker (Thermo Fisher Scientific, Waltham, MA, USA) and fluorophore-conjugated antibodies. (BioLegend,UK; Supplementary Table 1).

For T cells activation assay, following CD16/CD32 Fc receptor blocking, cells were stained with LIVE/DEAD Fixable Aqua Dead Cell stain kit (Thermo Fisher,) and fluorophore conjugated antibody against anti-CD8 antibody (BioLegend). Then, cells were fixed using IC Fixation Buffer and Permeabilizd using Intracellular Staining Perm Wash Buffer (BioLegend, UK) per manufacturer’s instruction, which then followed by the staining with fluorophore conjugated antibody against IFN-γ and Gramzyme-B (Biolegend, Supplementary Table 1).

Cells were incubated with antibodies on ice in the dark for 45 min and proceed to acquire flow cytometry data on a FACS Canto II using FACSDiva software (BD Biosciences, Franklin Lakes, NJ, USA) and analysed with FlowJo v.10 software (gaiting strategy is available in the Supplementary Figs. 2–5).

### Murine bone-marrow derived macrophages isolation

Primary murine bone marrow derived macrophages (BMDMs) were generated by flushing the femurs and tibias of C57BL/6 mice, followed by density gradient centrifugation and incubation with 10 ng/ml of murine M-CSF (Peprotech, AF-315-02) in DMEM supplemented with 10% FBS for 5 days.

### Generation of conditioned media

To prepare the tumour conditioned media (TCM), Py230 cells were seeded and let grown to 80% confluency in T75 flask. Cells were then washed with PBS and cultured with low serum (2% FBS) medium for 24 h. The TCM was harvested and centrifuged to remove the debris and dead cells.

To generate tumour educated macrophages conditioned media (TeMCM), BMDMs were treated with TCM (2% FBS) for 24 h, to obtain tumour educated macrophages (TEMs). TEMs were then treated with either hamster control IgG antibody anti-VISTA mAb treatment (20 µg/ml for both). After 24 h of antibody treatment, cells were washed with PBS and cultured with fresh medium for another 24 h. Finally, TeMCM were then collected and centrifuged to remove the debris and dead cells and proceed for subsequent experiment.

### T cell activation assay

Primary splenocytes were prepared from spleens of naïve C57BL/6 mice. Briefly, spleens were dissociated in MACS buffer and filtered through a 70 μm cell strainer to generate a single-cell suspension. After removing the red blood cells using RBC Lysis Buffer (BioLegend), splenocytes were then cultured in RPMI supplemented with 10% FBS.

For T cells activation where T cells co-cultured with TEMs and TENs, primary murine splenocytes were stimulated with Dynabeads Mouse T-Activator CD3/CD28 (Thermo Fisher), and then co-cultured with TEMs at a 20:1 ratio (Splenocytes:TEMs) with VISTA-blocking mAb or hamster control IgG (referred to IgG1 in this study, both at 20 μg/ml); PSGL-1-blocking antibody (BioXCell, BE0186) or rat control IgG (BioXCell, BE0088) (referred to as IgG2 in this study, 10μg/ml); VISTA-blocking mAb combine with PSGL-1 blocking mAb or IgG control (referred to as IgG1 + IgG2 in this study) as indicated. For examining whether the effects of TEMs exposed to VISTA-blocking antibody on CD8 + T cells is through cell-cell direct interaction or through soluble factors, activated splenocytes were cultured in TeMCM for 24 h. Cells were plated into 96-well plates and incubated at 37 °C for 24 h. Subsequently, cells were incubated with 1x Brefeldin A solution (eBioscience, 1:100) for 4–6 h prior to immunostaining and flow cytometry analysis as described above.

### Immunohistochemistry

Deparaffinization and antigen retrieval of formalin fixed paraffin embedded (FFPE) 4 μm human TNBC tissues sections were performed using the PT-Link system (Dako, Santa Clara, CA, USA), followed by the immunostaining using the Dako Envision Plus System. Tissue sections were incubated overnight at 4 °C with the primary antibodies (Supplementary Table 1), followed by incubation with HRP-conjugated secondary antibody for 30 min at room temperature. Staining was developed using Dako Liquid DAB+ Substrate Chromogen System (K3468, Agilent Technologies, Santa Clara, CA, USA) and counterstained with haematoxylin.

### Immunofluorescence

For FFPE human and mouse TNBC tissue staining, deparaffinization and antigen retrieval were performed using the PT-Link system (Dako), followed by permeabilization with 0.1% triton X-100 in PBS and blocked using 8% normal Donkey serum. Tissue sections were incubated at 4 °C with the primary antibodies (Supplementary Table 1). The following day, tissue sections were washed with PBS and incubated with 5 mg/ml nuclear dye DAPI (Thermo Fisher) and fluorescently labelled secondary antibodies (Supplementary Table 1) for 2 h at room temperature. Tissue sections were then washed with PBS and mounted onto coverslip using Dako Fluorescent mounting medium (S302380-2, Agilent). Imaging data were acquired using Axio observer Light microscope with the Apotome.2 (Zeiss) and analysed using Zen v.3.8 software (Zeiss). Positively stained cells were counted manually on the software (5–10 fields of views per tissue section).

### Quantitative real-time PCR

Total RNA was extracted from primary murine BMDMs or neutrophils using RNeasy Kit (QIAGEN,74106), the quality and quantity of extracted RNA was tested using Nanodrop spectrophotometer. Reverse transcription of RNA was performed using MMLV reverse transcriptase (Thermo Fisher, 28025013) according to the manufacturer’s instruction. Real-time PCR (Supplementary Table 2) was performed using 5x HOT FIREPol EvaGreen qPCR Mix Plus ROX (Solis Biodyne, 08-24-00020) on the AriaMX3005P RT-PCR system (Agilent). Relative mRNA expression levels were normalised to *Gapdh* expression according to the 2^−∆Ct^. Fold change was calculated using 2^−∆∆Ct^.

### Statistical analysis

Statistical analysis of experiments was performed in Graphpad Prism 8 (RRID:SCR_002798) using unpaired Student *t* test when comparing two groups or one-way ANOVA with Šidák multiple comparison tests when comparing multiple groups as indicated. Error bars represents SD when the means of individual data points are compared, or represents SEM when the means of biological replicates are being compared. A *P* < 0.05 was considered statistically significant.

### Institutional approval

All studies involving the use of Human TNBC tissue were approved by the University of Liverpool (Liverpool, UK) and National Research Ethics Service Committee North West-Greater Manchester (REC15/NW/0477). Human TNBC tissue were obtained from the Liverpool Tissue Bank and all patients provided written informed consents on approved institutional protocol.

## Results

### VISTA is predominantly expressed by macrophages and neutrophils in murine and human TNBC

**Fig. 1:**
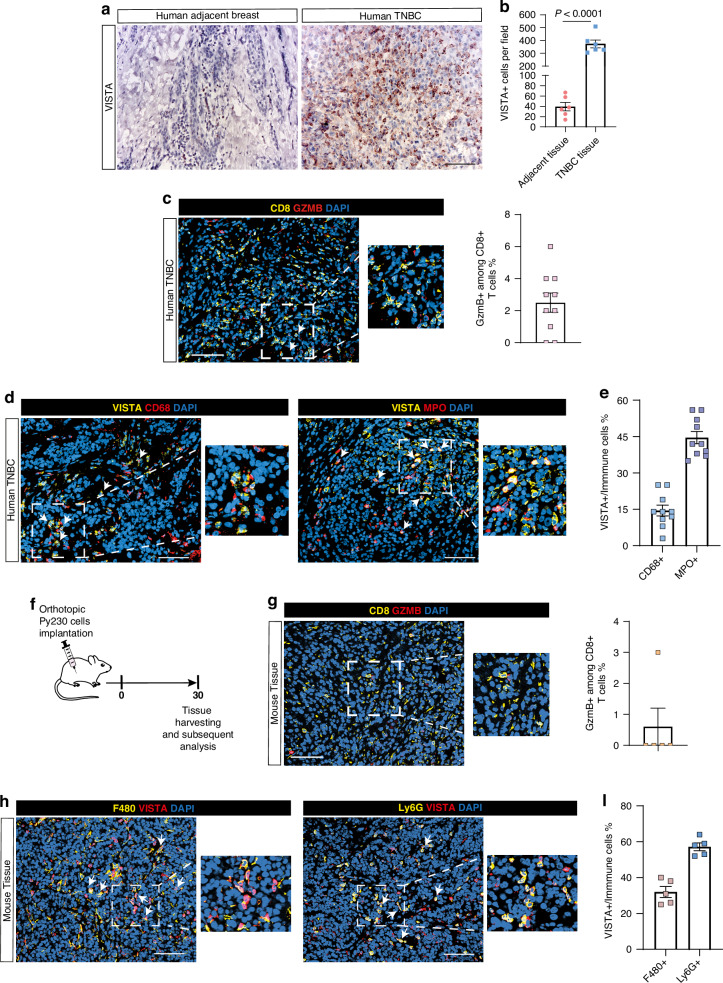
VISTA is predominantly expressed on macrophages and neutrophils in human and murine TNBC. **a**, **b** Representative IHC images and quantification of VISTA in serial sections from human TNBC and adjacent breast tissue. Scale bar, 50 µm. Data presented as mean number area. Error bars, mean ± SEM, *P* value, two-tailed unpaired *t*-test. **c** Representative IF images and quantification of cytotoxic GZMB + CD8 + T cells in human TNBC (*n* = 10 patient). Arrowheads, double positive cells. Scale bar, 50 µm. Quantification was done on 5–10 fields of view per patient. Error bars, mean ± SEM. Representative IF images (**d**) and quantification of VISTA + macrophages (CD68+) and neutrophils (MPO+) (**e**) in human TNBC tissue (*n* = 10 patients). Arrowheads, double positive cells. Scale bar, 50 µm. Quantification was performed on 5–10 fields of view per patient sample. Error bars, mean ± SEM. **f** Schematic of orthotopic implantation of Py230 breast cancer cells into the mammary fat pad of recipient syngeneic C57BL/6 mice. **g** Representative IF images and quantification of cytotoxic GZMB + CD8 + T cells in the murine primary tumours (*n* = 5 mice). Scale bar, 50 µm. Quantification was done on 3–5 fields of view per mouse. Error bars, mean ± SEM. Representative IF images (**h**) and quantification of VISTA+ macrophages (F4/80 +) and neutrophils (Ly6G +) (**I**) in murine TNBC tissue (*n* = 5 mice). Arrowheads, double positive cells. Scale bars, 50 µm. Quantification was performed on 3–5 fields of view per mouse (*n* = 5 mice). Error bars, mean ± SEM.

To investigate whether VISTA may be an important immune checkpoint regulator in TNBC, we examined its expression and cellular distribution in tissue sections obtained from resected samples of TNBC patients and adjacent non-involved breast tissue. Our result showed an elevated expression of VISTA in tumour tissue compared to adjacent non-involved breast tissue (Fig. 1a, b). Consistent with previous reports, TNBC tissues were infiltrated by functionally impaired CD8 + T cells, identified by the absence of Granzyme B (GzmB) expression (Fig. 1c). Next, using multiplex immunofluorescence staining, we aimed to characterise the main VISTA expressing cell subsets in TNBC. Our analysis revealed that VISTA is predominantly expressed by macrophages (CD68 +) and neutrophils (MPO +) (Fig. 1d, e), while cancer cells (CK11 +) and fibroblasts (αSMA +) showed lower to no expression of VISTA (Supplementary Fig. 1A, B).

To deepen our understanding of how VISTA affects anti-tumour immunity in TNBC, we utilised a preclinical mouse model previously described to recapitulate the progression of human TNBC [25, 26], in which Py230 breast cancer cells were orthotopically implanted into the mammary fat pad of recipient syngeneic mice for 30 days (Fig. 1f). Consistent with our observations in human TNBC, we found that most CD8 + T cells were functionally impaired, identifiable by the absence of GzmB (Fig. 1g). FOXP3+ regulatory T cells (Tregs) and CD45 + RO memory T cells were also identified within the tumour (Supplementary Fig. 1C, D). Furthermore, VISTA was expressed by both macrophages (F4/80 +) and neutrophils (Ly6G +) (Fig. 1h, i), confirming that the orthotopic implantation of Py230 cells efficiently recapitulates human TNBC.

Taken together, our data suggests that TNBC-infiltrating CD8 + T cells are largely dysfunctional, while macrophages and neutrophils are the main VISTA-expressing cell subsets.

### VISTA blockade enhances macrophages pro-inflammatory effect in a mouse model of TNBC

**Fig. 2:**
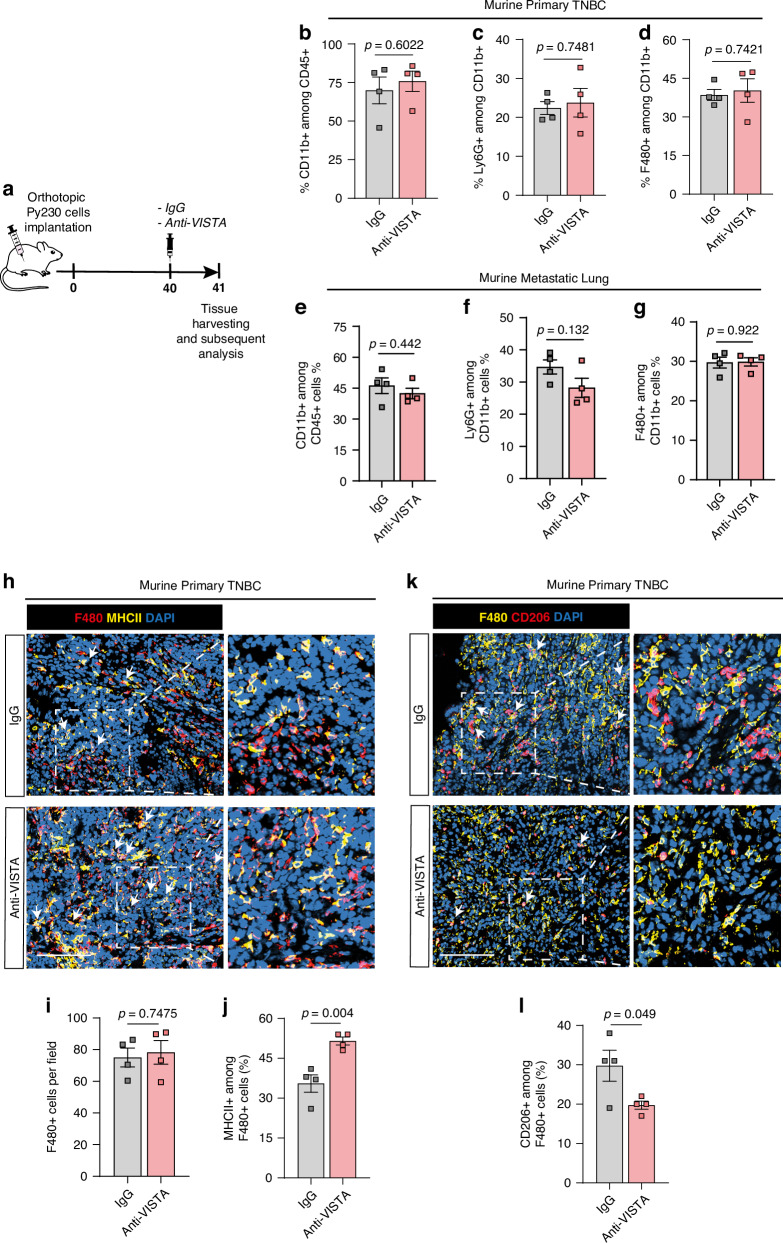
VISTA blockade regulates macrophages phenotype in murine TNBC tumours. **a** Schematic of orthotopic implantation of Py230 breast cancer cells into the mammary fat pad of recipient syngeneic C57BL/6 mice, mice were treated when tumours reached between 10 and 13 mm^2^, one dose i.p., with control IgG antibody and anti-VISTA blocking antibody (300 µg/mouse, *n* = *4* mice per group). **b**–**d** Flow cytometry analysis of CD11b+ cells (**b**), neutrophils (Ly6C + Ly6G +) (**c**), and macrophages (F480 +) (**d**) and in the primary TNBC tumours (*n* = 4 mice/group). Error bars, mean ± SEM. *P* value, two-tailed unpaired *t*-test. **e**–**g** Flow cytometry analysis of CD11b+ cells (**e**), macrophages (F480 +) (**f**) and neutrophils (Ly6C + Ly6G +) (**g**) in the lung metastasis from mice treated with IgG control and anti-VISTA antibody (*n* = 4 mice/group). Error bars, mean ± SEM. *P* value, two- tailed unpaired *t*-test.  **h**–**j** Representative IF images (**h**) and quantification of total (**i**) and MHCII+ macrophages (F480 +) (**j**) in the murine primary tumours from mice treated with IgG control antibody and anti-VISTA blocking antibody. Arrowheads, double positive cells, Scale bars, 50 µm. Quantification was performed on 3–5 fields of view per mouse (*n* = 4 mice/group). Error Bars, mean ± SEM. *P-*value, two-tailed unpaired *t*-test. **k**, **l** Representative IF images (**k**) and quantification of CD206+ macrophages (F480 +) (**l**) in the murine primary tumours from mice treated with IgG control antibody and anti-VISTA blocking antibody. Arrowheads, double positive cells, Scale bars, 50 µm. Quantification was performed on 3–5 fields of view per mouse (*n* = 4 mice/group). Error Bars, mean ± SEM. *P* value, two-tailed unpaired *t*-test.

VISTA can affect anti-tumour immunity through various mechanisms, including regulating the composition and function of myeloid cells within the TME [19, 27, 28]. Accordingly, we next aimed to elucidate the functional role of VISTA by pharmacological blockade of VISTA on anti-tumour immunity in TNBC by using the VISTA-specific blocking antibody 13F3 in-vivo. TNBC is highly aggressive and readily metastasises to the lung [29], thus we orthotopically implanted Py230 breast cancer cells into the mammary fat pad of WT C57BL/6 mice and prolonged tumour growth to allow lung metastases in this model (Fig. 2a). At day 40, mice were administered a single dose of VISTA-neutralising antibody and sacrificed at day 41 for further interrogation. We previously observed that macrophages and neutrophils expressed VISTA, therefore we focussed our further analysis on these cell populations. Flow cytometry analysis revealed no significant differences in the number of myeloid cells (CD11b +), neutrophils (Ly6G +), or macrophages (F4/80 +), between IgG control and anti-VISTA treated groups in both primary tumours (Fig. 2b–d, Supplementary Fig. 2A) and metastatic lungs (Fig. 2e–g, Supplementary Fig. 2B). These results were further supported by immunofluorescent staining of the tissue (Fig. 2h, i and Supplementary Fig. 2C–F). Given that VISTA may have a role in regulating macrophages functions as previously reported [13], we next examined MHCII+ and CD206+ macrophages using immunostaining. As expected, we found that macrophages infiltrating tumours from anti-VISTA treated mice showed significantly elevated levels of MHCII expression (Fig. 2h, j) and reduced levels of CD206 expression (Fig. 2k, l), suggesting a functional shift in macrophages towards tumour-suppressing phenotype. In contrary, the numbers of MHCII + CD11C+dendritic cells remained unchanged in response to VISTA blockade (Supplementary Fig. 3A, B).

In metastatic lungs, despite no significant changes on MHCII expression (Supplementary Fig. 2E, G), we observed a notable decrease in CD206 expression among macrophages (Supplementary Fig. 2H, I) from anti-VISTA treated mice compared to the controls, indicating a potential shift in their phenotype. In sentinel lymph nodes, VISTA blockade did not significantly affect the presence of MHCII + CD11C+ dendritic cells (Supplementary Fig. 3C, D).

Together, these findings suggest that VISTA-blockade induces a functional shift in macrophages, at both the primary and metastatic site, towards a tumour-suppressive phenotype.

### Blocking VISTA regulates macrophages phenotypes in-vitro

**Fig. 3:**
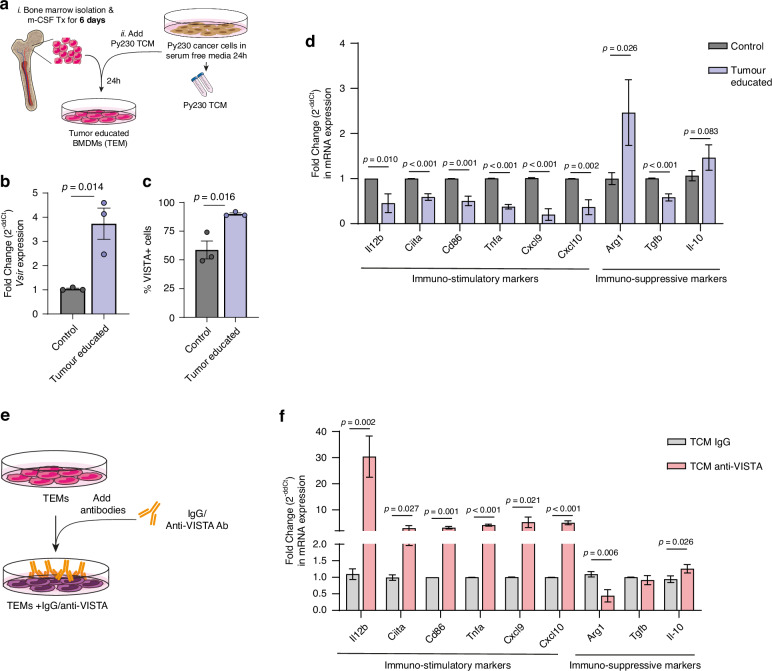
VISTA blockade regulates macrophage phenotype in vitro. **a** Schematic of primary bone-marrow derived macrophages isolation and exposure to Py230 TCM for 24 h followed by incubation with anti-VISTA blocking antibody. **b** qPCR analysis of *Vsir* expression in tumour educated macrophages (TEMs) (24 h, *n* = 3 biological replicates). Error Bars, mean ± SEM. *P*-value, two-tailed unpaired *t*-test. **c** Flow cytometry analysis of VISTA + among TEMs (F480 +) (*n* = 3 biological replicates). Error Bars, mean ± SEM. *P* value, two-tailed unpaired *t*-test. **d** qPCR analysis of genes (*Il12b*, *Ciita, Cd86, Tnfa, Cxcl9, Cxcl10, Arg1, Il10, Tgfb1*) expression in TEMs (*n* = 3 biological replicates). Error Bars, mean ± SEM. *P* value, two-tailed unpaired *t*-test. **e** Schematic of TEMs incubation with IgG control and anti-VISTA blocking antibody. **f** qPCR analysis of genes (*Il12b*, *Ciita, Cd86, Tnfa, Cxcl9, Cxcl10, Arg1, Il10, Tgfb1*) expression in TEMs treated with IgG control and anti-VISTA antibody (*n* = 3 biological replicates). Error Bars, mean ± SEM. *P* value, two-tailed unpaired *t*-test.

Having observed that VISTA blockade promotes an immunostimulatory phenotype of macrophages in-vivo, we next aimed to further explore the specific functions of VISTA on macrophages, in-vitro. First, we generated primary bone-marrow derived macrophages and exposed them to tumour conditioned media (TCM) from Py230 cells to generate tumour-educated macrophages (TEMs) (Fig. 3a). Consistent with an upregulation of VISTA in TNBC tissue compared to adjacent normal tissue, exposure of macrophages to TCM induced a significant upregulation of *Vsir* (encoding VISTA) (Fig. 3b), and VISTA protein expression on the cell surface (Fig. 3c) on tTEMs, suggesting that cancer cells regulate VISTA expression on macrophages. To interrogate the phenotypical status of VISTA+ TEMs, we further assessed a number of immunosuppressive (*Arg1*, *Il-10*, *Tgfb1*) and pro-inflammatory (*Il12b, Ciita, Cd86, Tnfα, Cxcl9, Cxcl10*) gene markers, revealing the induction of an overwhelmingly immunosuppressive phenotype (Fig. 3d).

To further explore which factors within the TCM might drive the upregulation of VISTA on macrophages, we performed qPCR analysis to assess the expression of the cytokines, chemokines and growth factors: *Inf-g, Il-4, Il-6 Il-10, Tnfa, and Vegfa* in Py230 cells. These factors were assessed as they are cancer cell-derived mediators known to regulate immune checkpoint expression, including VISTA [30–34]. The qPCR analyses revealed that all these factors are expressed by Py230 cells, albeit in relatively low levels, when compared to *Gapdh* (supplementary Fig. 4A). These data suggest that any of these factors could potentially increase VISTA levels on macrophages in vivo when they are in proximity to Py230 cancer cells.

A recent study suggested that IL-4 is a potent inducer of the VISTA^+^ phenotype in macrophages [34], therefore, we treated macrophages with IL-4 or TCM and found that IL-4 treatment alone resulted in an increased expression of VISTA and CD206 (Supplementary Fig. 4B, C), however TCM treatment further upregulated the expression of VISTA on macrophages (Supplementary Fig. 4C). These findings suggest that IL-4 is secreted by Py230 and can upregulate VISTA expression on macrophages but that there are other cancer secreted factors that can also upregulate VISTA expression.

Next, to investigate the effect of VISTA blockade on macrophages, we treated TEMs with either IgG control or anti-VISTA antibody and interrogated the expression of our previously defined immunosuppressive and pro-inflammatory gene signatures (Fig. 3e). Consistent with our in vivo results, TEMs treated with anti-VISTA neutralising antibody showed a significant increase in the expression of pro-inflammatory genes including *Il12b, Ciita, Cd86, Tnfα, Cxcl9 and Cxcl10* (Fig. 3n–s), accompanied by a decrease in the expression of the immunosuppressive gene *Arg1*, but not *Il-10* or *Tgfb1*, compared to those treated with IgG control (Fig. 3f). Taken together, our results suggest that VISTA regulates an immunosuppressive macrophage phenotype, and that blockade of VISTA with neutralising antibody is sufficient to restore an inflammatory phenotype that is associated with tumour-suppressive functions.

### Blocking VISTA on macrophages reverses its immunosuppressive activity on CD8 + T cells

**Fig. 4:**
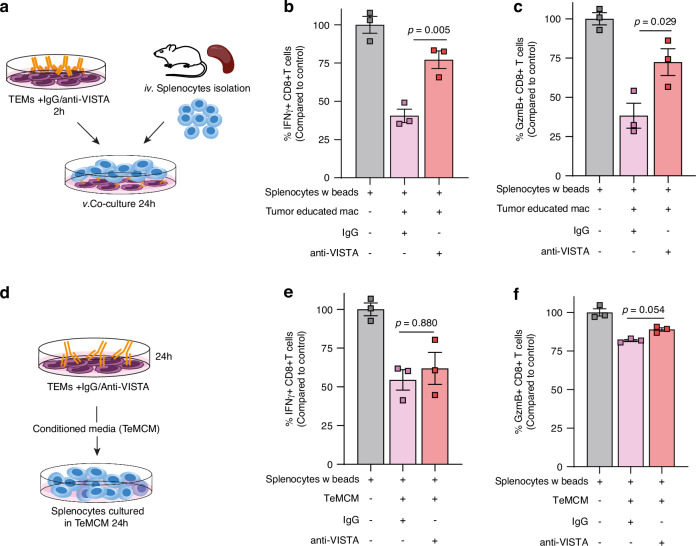
VISTA blockade on TEMs restores T cell activation. **a** Schematic of T cells activation assay, primary bone-marrow derived macrophages exposed to Py230 TCM for 24 h followed by pre-incubation with anti-VISTA blocking antibody for 2 h, and co-cultured with primary splenocytes stimulated with anti-CD3/CD28-coupled Dynabeads. **b**, **c** Relative activation levels of CD8 + T cell, assessed as the percentages of IFN-γ + CD8+ (**b**) and GzmB+ CD8+ (**c**) T cells, co-cultured with TEMs in the presence of IgG control and anti-VISTA blocking antibody (*n* = 3 biological replicates). Error Bars, mean ± SEM. *P* value, One-way ANOVA with Šidák multiple comparison test. **d** Schematic of T cells activation assay in TeMCM collected from TEMs exposed to anti-VISTA antibody. **e**, **f** Relative activation levels of CD8 + T cell, assessed as the percentages of IFN-γ + CD8+ (**e**) and GZMB + CD8+ (**f**) T cells, primary splenocytes stimulated with anti-CD3/CD28-coupled Dynabeads cultured in TeMCM (*n* = 3 biological replicates). Error Bars, mean ± SEM. *P* value, One-way ANOVA with Šidák multiple comparison test.

CD8 + T cells are central mediators of anti-tumour immunity, and immunosuppressive macrophages are known to potently suppress CD8 + T cell functions [35, 36]. Given that VISTA blockade was sufficient to repolarise TEMs, we hypothesised that the acquisition of an immunostimulatory phenotype could dampen the suppression of CD8 + T cells, restoring their activation and cytotoxic functions. To examine the effect of VISTA blockade on the immunosuppressive functions of macrophages, TEMs treated with either IgG control or anti-VISTA antibody were co-cultured with CD8 + T cells generated from stimulated primary splenocytes (Fig. 4a). Co-culture experiments reveal that TEMs treated with IgG control exert a potent and suppressive effect on CD8 + T cell function, determined by suppressed IFNγ and GzmB expression (Fig. 4b, c and supplementary Fig. 4A). Notably, pre-treating TEMs with anti-VISTA neutralising antibody was sufficient to partially reverse the suppression of CD8 + T cells.

Given our observation that VISTA blockade induced upregulation of secreted immunostimulatory cytokines, we next exposed stimulated primary splenocytes to conditioned media (CM) from TEMs pre-treated with IgG control or anti-VISTA neutralising antibody (Fig. 4d). Interestingly, while exposure to TeMCM was less suppressive to CD8 + T cell function than direct co-culture, we observed no significant difference in IFN-γ or GzmB expression in CD8 + T cells exposed to TeMCM pre-treated with either IgG control of anti-VISTA neutralising antibody (Fig. 4e, f). Together, these results suggest that the pre-treatment of TEMs with anti-VISTA neutralising antibody restores CD8 + T cell function not through soluble factors, but through direct cell-cell interaction.

PSGL-1, expressed by T cells, has been reported to interact with VISTA and mediate T cell dysfunction [37, 38]. A previous study showed that a VISTA blocking antibody, which inhibits the VISTA/PSGL-1 interaction, results in increased production of IFN-γ by T cells co-cultured with VISTA expressing cells [37]. Therefore, we aimed to elucidate whether the immunosuppressive effect exerted by VISTA expressing TEMs on CD8 + T cells is mediated through the interaction between VISTA and PSGL-1 on T cells. To investigate this, we co-cultured TEMs with stimulated splenocytes in the presence, or absence, of anti-VISTA, anti-PSGL-1 and the two combined (Supplementary Fig. 4B). Our analysis reveals that the treatment with anti-VISTA antibody alone or in combination with PSGL-1 reverses the TEMs mediated suppression of CD8 + T cell. However, anti-PSGL-1 alone could not reverse the suppression on CD8 + T cell activation (Supplementary Fig. 4C, D), suggesting that VISTA expressed on macrophages suppresses CD8 + T cells in a PSGL-1 independent manner.

Collectively, these findings suggest that tumour educated macrophages suppress CD8+ T cell function via VISTA through cell-cell interaction, which is independent of PSGL-1.

### VISTA blockade enhances CD8 + T cells mediated anti-tumour immune response in a mouse model of TNBC

**Fig. 5:**
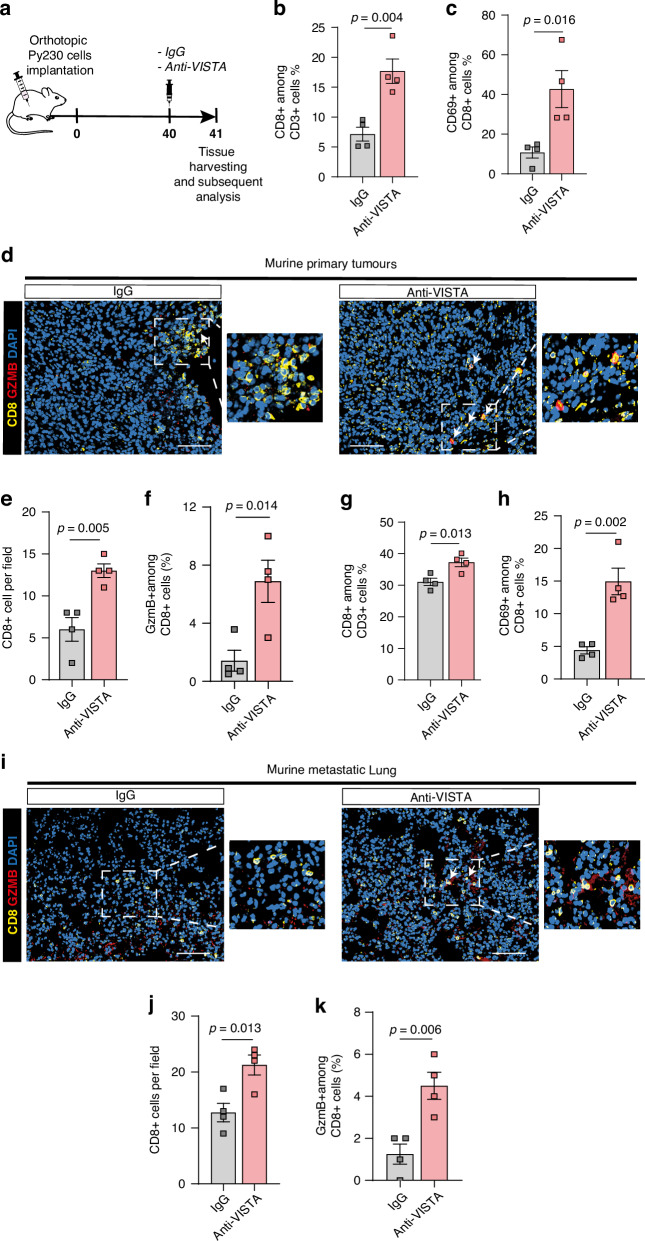
VISTA blockade enhances CD8 + T cells mediated anti-tumour immune response in-vivo. **a** Schematic of orthotopic implantation of Py230 breast cancer cells into the mammary fat pad of recipient syngeneic C57BL/6 mice, mice were treated when tumours reached between 10 and 13 mm^2^, one dose i.p., with control IgG antibody and anti-VISTA blocking antibody (300 µg/mouse, *n* = 4 mice per group). **b**, **c** Flow cytometry analysis of CD3 + CD8 + T cells (**b**) and CD69 + CD8 + T cells (**c**) in primary tumours (*n* = 4 mice per group). Error bars, mean ± SEM. *P* value, two-tailed unpaired *t*-test. **d**–**f** Representative IF images (**d**) and quantification of total (**e**) and cytotoxic GZMB+ (**f**) CD8 + T cells in the murine primary tumours. Scale bars, 50 µm. mean ± SEM. *P* value, two-tailed unpaired *t*-test. g-h Flow cytometry analysis of CD8 + T cells (**g**) and CD69 + CD8 + T cells (**h**) in metastatic lung. Error bars, mean ± SEM. *P* value, two-tailed unpaired *t*-test. **i**–**k** Representative IF images (**i**) and quantification of total (**j**) and cytotoxic GZMB+ (**k**) CD8 + T cells in the metastatic lung tissue. Scale bars, 50 µm. Quantification was performed on 3–5 fields of view per mouse tissue (*n* = 4 mice/group). Error Bars, mean ± SEM. *P-* value, two-tailed unpaired *t*-test.

Our in-vitro experiments demonstrated that blockade of VISTA on macrophages dampens their suppressive effect on CD8 + T cells activation, suggesting a potential mechanism by which tumour infiltrating VISTA+ macrophages regulate anti-tumour immunity at both the primary and metastatic site of TNBC. To validate this further in-vivo, we revisited our preclinical mouse model of spontaneously metastasising TNBC to evaluate the effects of VISTA blockade on CD8 + T cell infiltration and function (Fig. 5a).

Flow cytometry analysis revealed a significant increase in the total number of CD8 + T cells (Fig. 5b, Supplementary Fig. 5A) within primary tumours following VISTA blockade, compared to the IgG-treated group. Moreover, the proportion of CD69 + CD8 + T cells (Fig. 5c) was significantly higher in the anti-VISTA treated group, indicating enhanced CD8 + T cell activation within the TME. In support of these results, IF staining of primary tumours revealed a significant increase in total CD8 + T cell numbers (Fig. 5d, e), and cytotoxic GzmB+CD8 + T cells (Fig. 5f) upon VISTA blockade suggesting enhanced cytotoxic activity. To address whether these immune changes following VISTA blockade affected tumour growth, we assessed markers of tumour proliferation and apoptosis. As expected, IHC analysis of primary tumours revealed no significant differences in Ki67 or cleaved caspase-3 between the anti-VISTA-treated and IgG-treated groups (Supplementary Fig. 5C, D).

Next, to evaluate the systemic effects of VISTA blockade, we analysed the lung tissue. Flow cytometry analysis of metastatic lungs also revealed a significant increase in the total number of CD8 + T cells (Fig. 5g, Supplementary Fig. 5B) upon VISTA blockade, compared to IgG control mice, which coincided with a significant increase in the number of activated CD69 +  CD8 + T cells (Fig. 5h). Consistent with these findings, IF staining revealed a significant increase in both CD8 + T cells and GzmB+CD8+ T cells (Fig. 5i–k), indicative of enhanced cytotoxic activity. To further assess the impact of VISTA blockade on metastatic burden, we performed CK19 staining to identify metastatic foci. Although we observed a reduction in CK19+ metastatic foci in the anti-VISTA-treated group compared to IgG controls, this difference did not reach statistical significance (Supplementary Fig. 5E, F).

Taken together, our results support that reprograming of macrophages by VISTA neutralising antibody dampens the suppression of CD8 + T cells, resulting in enhanced CD8+ infiltration, activation, and cytotoxicity. These findings indicate a restored anti-tumour immune response at both the primary and lung metastatic site. Our results highlight the potential systemic therapeutic benefit of targeting VISTA+ macrophages in advanced TNBC.

## Discussion

In this study, we conducted a comprehensive analysis to assess how VISTA affects the anti-tumour immune response in TNBC. Using tumour tissue samples obtained from patients with TNBC, we observed increased VISTA expression in comparison to non-involved breast tissue, with macrophages and neutrophils being the dominant VISTA expressing cell population, while tumour infiltrating CD8 + T cells are largely dysfunctional. Similarly, in our mouse model of breast cancer, we found VISTA was abundantly expressed by tumour infiltrating macrophages and neutrophils, while CD8 + T cells are largely dysfunctional. Meanwhile, blockade of VISTA with neutralising antibody reprogrammed tumour-associated macrophages into a pro-inflammatory and classically tumour-suppressive phenotype and restored CD8 + T cell activation and function in-vitro and in-vivo, both at the primary and lung metastatic site. These findings suggest a potential therapeutic benefit for targeting VISTA in patients with TNBC that exhibit poor response to standard ICI therapy.

There has been an increasing interest in VISTA as an immunotherapeutic target due to its broad expression on multiple immune cells, its role in suppressing immune responses, and more importantly, its non-overlapping function with PD-1/PD-L1 or CTLA-4 [20, 39]. Functional blockade or depletion of VISTA was previously showed various effect on immune cells in multiple mouse model, including the increase in the abundance of myeloid cells exhibiting a pro-inflammatory phenotype, decrease in the frequency of immunosuppressive regulatory T cells, enhanced infiltration and activation of CD8 + T cell [19, 27].

In our study, VISTA was predominantly expressed by macrophages and neutrophils in TNBC tissue. Our results indicate that IL4 and possibly other factors secreted by Py230 cancer cells upregulate VISTA expression on macrophages. These findings suggest that while cancer cell derived IL-4 contributes to VISTA upregulation on macrophages, the full effect of the TCM likely involves additional factors that act synergistically to enhance VISTA expression. For instance, in a mouse model of bladder cancer, TGFβ has been recently shown to correlate with high VISTA expression [13], while the TGF-β-Smad-3 signalling pathway has previously been shown to positively regulate VISTA expression in human T lymphocytes [40]. Whether TGF-β or other tumour-secreted factors regulate VISTA in TNBC remains to be determined.

The immediate effect of blocking VISTA with 13F3 monoclonal antibody led to a significant shift in macrophage phenotype towards a pro-inflammatory state, shown by increased levels of MHCII expression and reduced level of CD206 expression on tumour associated macrophages. Further, blocking VISTA on TNBC-educated primary bone-marrow derived macrophages in vitro resulted in a significant increase in the expression of pro-inflammatory genes, including *Il12*, *Ciita*, *Cd86*, *Tnfα*, *Cxcl9*, *Cxcl10* and a concurrent decrease in the expression of the immunosuppressive factor *Arg1, but not Il10 or Tgfb*. These observations strongly align with other studies suggesting a regulatory role of VISTA in modulating macrophage phenotype within the tumour microenvironment [22, 41, 42]. Cytokines such as *Il12*, *Ciita*, *Cd86*, *Tnfα* are well-known for their role in promoting T cells mediated anti-tumour immune response. Heightened expression of these cytokines can contribute to the formation of highly immunostimulatory environment conductive to effective anti-tumour immune response [43, 44]. *Cxcl9* and *cCxcl10* play a crucial role in the recruitment of cytotoxic CD8 + T cell and Th1 CD4 + T cell into the tumour site, and are generally associated with better prognosis and enhanced response to Immunotherapy [45].

Tumour-associated macrophages represent a major component of tumour-infiltrating immune cells in breast tumours/TNBC and contribute to the formation of immunosuppressive TME [46, 47]. The ability of macrophages to switch from pro-inflammatory to immunosuppressive phenotypes play a critical role in shaping the TME and influence the induction of an effective anti-tumour immune response, due to their regulatory role in T cell function. Consistent with these observations, our results supported that TEMs potently suppress CD8 + T cell cytotoxicity, both directly and indirectly. However, VISTA blockade on TEMs partially mitigated CD8 + T cell suppression, as evident by heightened IFNγ and GzmB expression, only under direct cell-cell contact.

Under acidic conditions, VISTA has previously been shown to directly bind PSGL-1 on T cells, potently suppressing T cell toxicity [37]. Our results, under standard conditions, revealed that PSGL-1 blockade was not capable of inhibiting macrophage-mediated suppression of CD8 + T cell cytotoxicity, suggesting that VISTA can also mediate T cell suppression directly, via a mechanism independent of PSGL-1. Further investigation is necessary to determine the underlying molecular mechanism of VISTA-mediated T cell suppression under direct cell-cell contact.

While VISTA blockade significantly enhanced CD8 + T cell activation and increased macrophage pro-inflammatory cytokine expression, it did not significantly reduce primary tumour growth or metastatic burden, although we can already appreciate a trend towards a reduced metastatic burden in the lungs of mice treated with only one dose of anti-VISTA antibody. This outcome was anticipated, given the single dose and short treatment duration, which may not have allowed sufficient time to affect tumour progression. However, the main aim of this study was to understand whether and how VISTA directly regulates the immune response. Our study provides mechanistic understanding of how VISTA expression on macrophages regulates CD8+ T cell function suggesting VISTA as a promising therapeutic target worth further exploring in TNBC and possibly other cancer types too. Future pre-clinical and clinical studies evaluating the therapeutic benefit of blocking VISTA in combination with chemotherapy and/or other immune checkpoint inhibitors in pre-clinical tumour models will reveal the effect of blocking VISTA in tumour growth, metastasis and survival. Notably, the non-overlapping functions of VISTA and PD-1/PD-L1, along with their frequent co-expression, suggest that combining VISTA inhibitors with ICIs could synergistically enhance anti-tumour immune responses. This approach could address resistance mechanisms that limit the efficacy of current therapies, particularly in TNBC.

It is important to note that we also observed a significant expression of VISTA on neutrophils in TNBC, but did not extensively investigate their role. Recent findings have also shown the potential role of VISTA in regulating the expression of inflammatory cytokines and chemokines by neutrophils stimulated with LPS in-vitro [22], as well as significant changes in neutrophil infiltration and suppressive phenotype in response to VISTA blockade in a pre-clinical mouse model of colon cancer [21]. In contrast, in our TNBC model, we did not observe significant changes in the number of infiltrating neutrophils following VISTA blockade, further exploration is necessary to determine whether pharmacological blockade of VISTA on neutrophils is also capable of reverting their suppressive phenotype in TNBC.

In summary, our study highlights the important role of VISTA-expressing macrophages in regulating anti-tumour immunity in primary and metastatic TNBC. Inhibition of VISTA leads to increased infiltration and activation of CD8 + T cells and re-programmes macrophages towards an immune-stimulatory phenotype, representing a promising therapeutic strategy for enhancing anti-tumour immune response in patients with TNBC.

## Supplementary information

Supplementary Figures and Figure legends

Supplementary Figure 1

Supplementary Figure 2

Supplementary Figure 3

Supplementary Figure 4

Supplementary Figure 5

Supplementary tables

## Data Availability

Data generated in this study are included within the supplementary material. All other raw data generated in this study are available upon request.
