# Supplementary material for: Macrophages suppress CD8 + T cell cytotoxic function in triple negative breast cancer via VISTA
Source: Br J Cancer. 2025 May 2;133(1):40–51. doi: 10.1038/s41416-025-03013-5 (PMC12238233; doi:10.1038/s41416-025-03013-5)
Supplement: Supplementary file 1 — Supplementary Figures and Figure legends [file 41416_2025_3013_MOESM1_ESM.docx]

**Supplementary Figures**

**
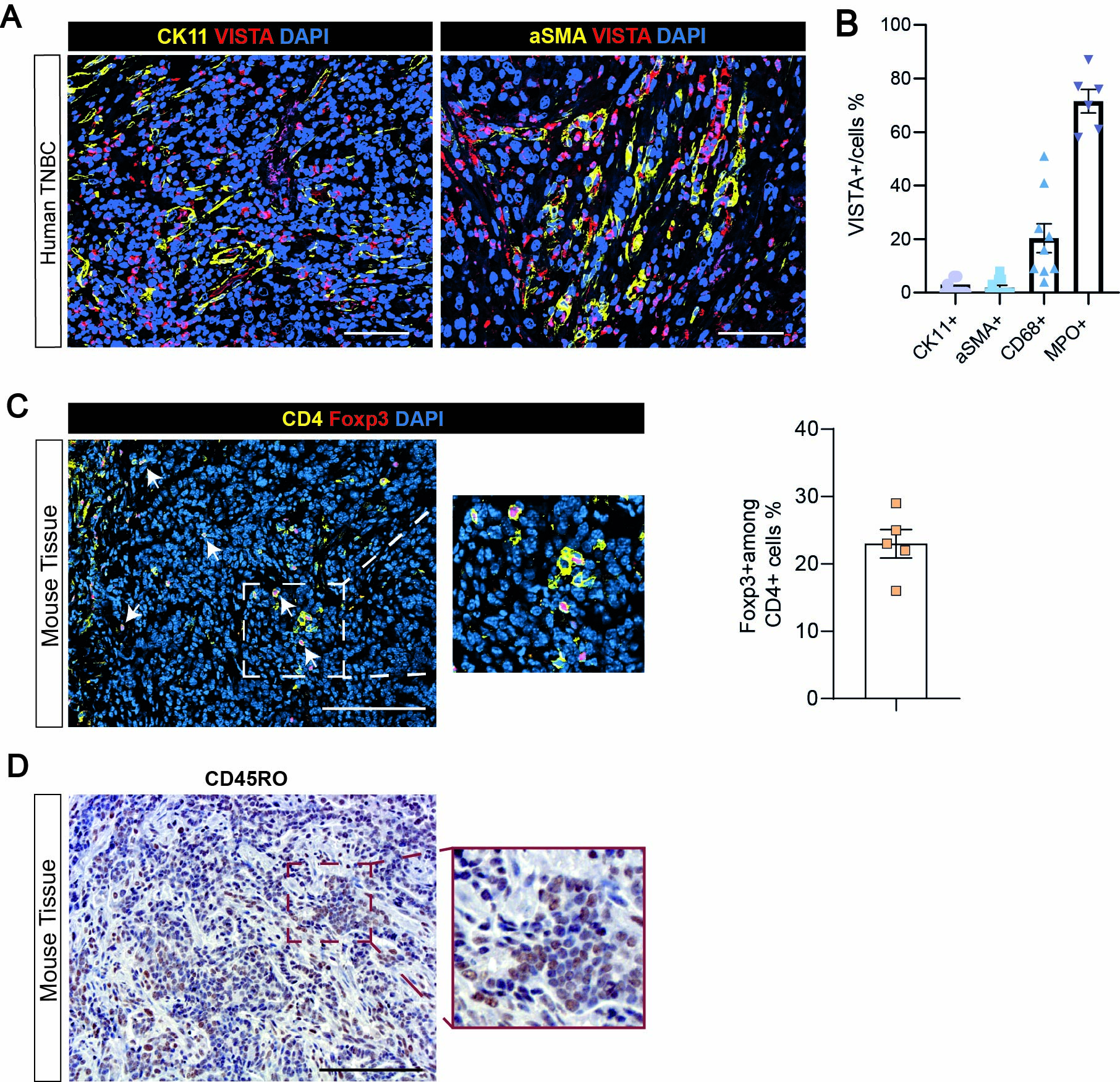
**

**Supplementary Figure-1 Cancer cells and tumour infiltrating fibroblasts do not express VISTA**

**(A),** Representative IF images of VISTA + cancer cells (CK11+) and VISTA + fibroblasts (αSMA+) in the human TNBC tissue. Scale bar, 50 µm.

**(B),** Quantification of VISTA+ cells among CK11+, αSMA+, CD68+ and MPO+ cells. Quantification was done on 5-10 fields of view per patient sample (n=1).

**(C),** Representative IF images and quantification of CD4+FOXP3 staining, showing the presence of regulatory T cells (Tregs) in the mouse TNBC tissue (n=5). Scale bars, 50 µm. Quantification was done on 3-5 fields of view per mouse. Error bars, mean ± SEM.

**(D),** Representative IHC images of CD45RO staining in the mouse TNBC tissue. Scale bars, 50µm.


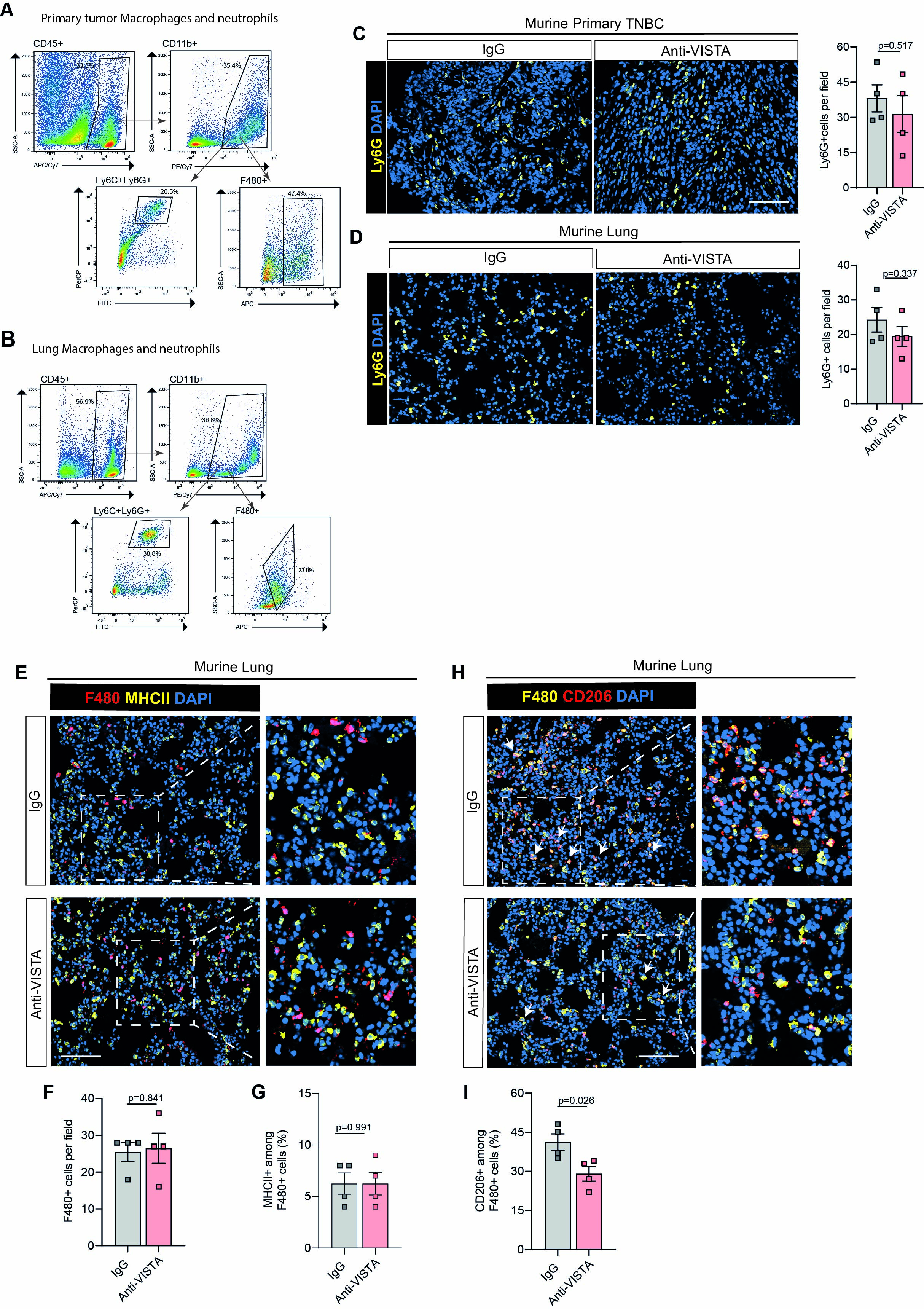


**Supplementary Figure-2 VISTA blockade regulates macrophage phenotypes in murine metastatic lung tissue**

**(A-B),** Gating strategy for flow cytometry analysis of myeloid cells (CD11b+), macrophages (F480+) and neutrophils (Ly6C+Ly6G+) in the primary TNBC tumours (**A**) and metastatic lung (**B**).

**(C-D),** Representative IF images and quantification of Ly6G+ neutrophils in primary tumours (**C**) and metastatic lung (**D**) from mice treated with IgG control antibody and anti-VISTA blocking antibody. Scale bars, 50 µm. Quantification was done on 3-5 fields of view per mouse. Error bars, mean ± SEM. *P*-value, two-tailed unpaired *t*-test.

**(E-G),** Representative IF images (**E**) and quantification of total (**F**) and MHCII+ (**G**) macrophages (F480+) in the murine primary tumours from mice treated with IgG control antibody and anti-VISTA blocking antibody. Arrowheads, double positive cells, Scale bars, 50 µm. Quantification was performed on 3-5 fields of view per mouse (*n*=4 mice/group). Error Bars, mean ± SEM. *P*-value, two-tailed unpaired *t*-test.

**(H-I),** Representative IF images (**H**) and quantification of CD206+ (**I**) macrophages in the murine primary tumours from mice treated with IgG control antibody and anti-VISTA blocking antibody. Arrowheads, double positive cells, Scale bars, 50 µm. Quantification was performed on 3-5 fields of view per mouse (*n*=4 mice/group). Error Bars, mean ± SEM. *P*-value, two-tailed unpaired *t*-test.


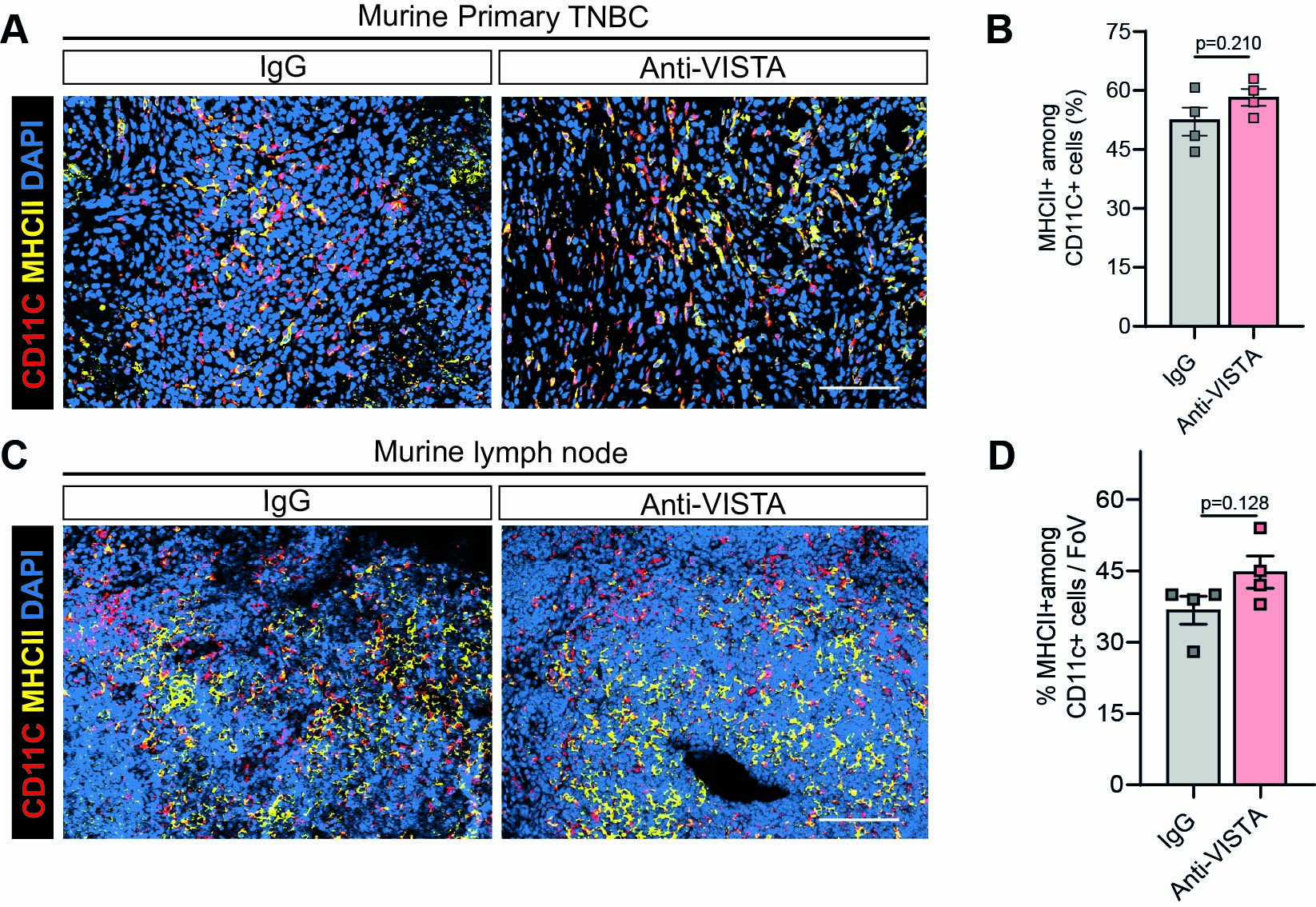


**Supplementary Figure-3 VISTA blockade did not affect MHCII expression in dendritic cells in primary tumour and sentinel lymph nodes**

**(A-B),** Representative IF images (**A**) and quantification of MHCII+ (**B**) cells among dendritic cells (CD11c+) in the primary tumours from mice treated with IgG control antibody and anti-VISTA blocking antibody. Scale bars, 50 µm. Quantification was performed on 3-5 fields of view per mouse (*n*=4 mice/group). Error Bars, mean ± SEM. *P*-value, two-tailed unpaired *t*-test.

**(C-D)**, Representative IF images (**C**) and quantification of MHCII+ dendritic cells (CD11c+) in the lymph nodes (**D)** from mice treated with IgG control antibody and anti-VISTA blocking antibody. Scale bars, 50 µm. Quantification was performed on 3-5 fields of view per mouse (*n*=4 mice/group). Error Bars, mean ± SEM. *P*-value, two-tailed unpaired *t*-test.


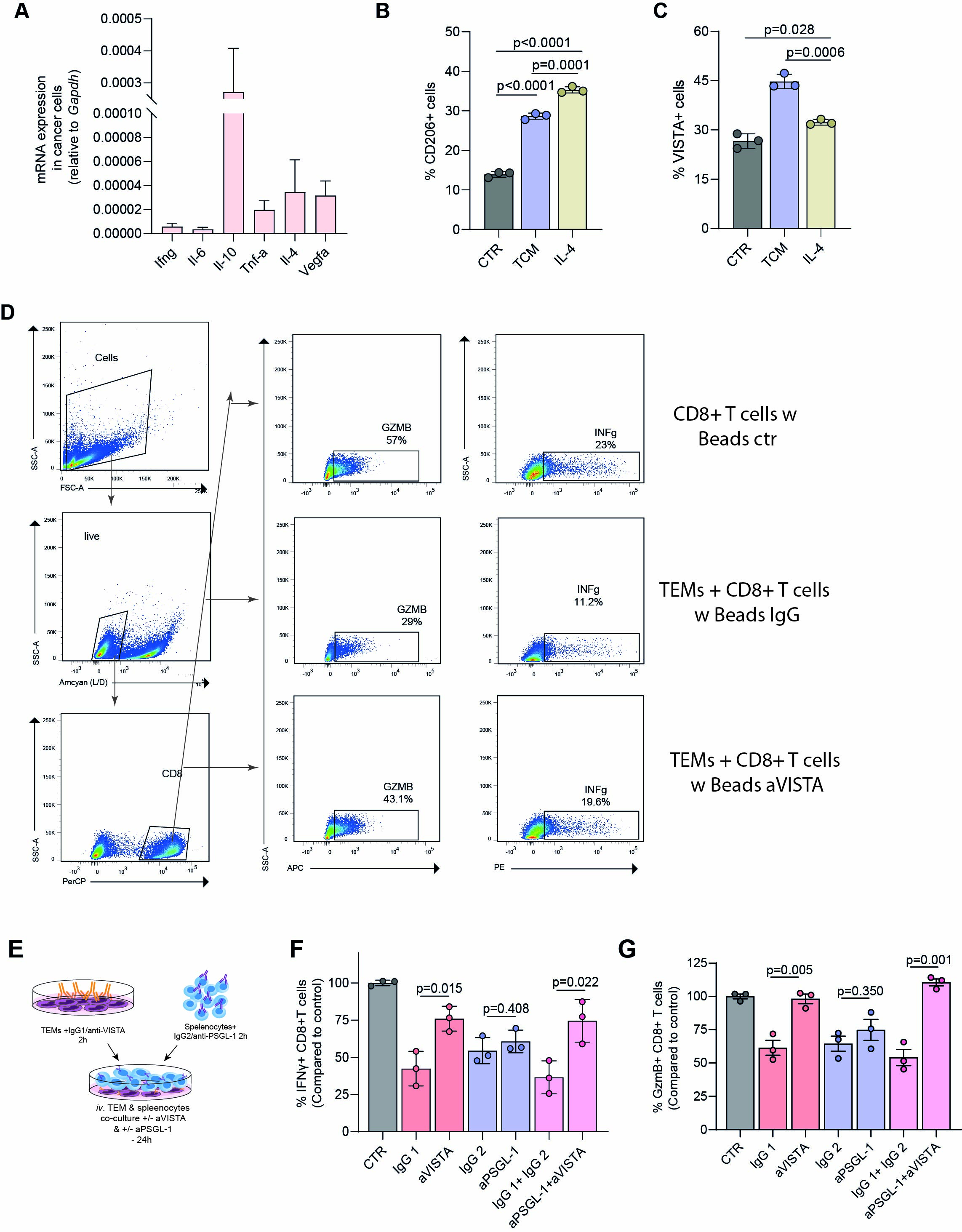


**Supplementary Figure-4 Assessment of T cells activation upon co-culture with macrophages *in-vitro***

**(A),** Relative mRNA expression of cytokines and chemokines (*Ifng, Il4, Il6, Il10, Tnfα, and Vegfa*) in Py230 cells as determined by qPCR. Data are normalized to *Gapdh*. Error bars represent mean ± SEM (n=3 biological replicates).

**(B),** FACS quantification of %CD206+ macrophages after treatment with TCM or IL-4 compared to untreated controls (CTR) by flow cytometry. Data are presented as the mean ± SEM (n=3 biological replicates). Statistical significance was calculated using one-way ANOVA with Šidák multiple comparison test.

**(C),** FACS quantification of %VISTA+ macrophages after treatment with TCM or IL-4 compared to untreated controls (CTR) by flow cytometry. Data are presented as the mean ± SEM (n=3 biological replicates). Statistical significance was calculated using one-way ANOVA with Šidák multiple comparison test.

**(D),** Gating strategy for flow cytometry analysis of T cells activation measured by the percentage of IFN-γ+CD8+ and GZMB+CD8+ T cells, stimulated with anti-CD3/CD28-coupled Dynabeads and co-cultured with TEMs in the presence of IgG control and anti-VISTA antibody.

**(E),** Schematic of T cells activation assay, TEMs co-cultured with primary splenocytes stimulated with anti-CD3/CD28-coupled Dynabeads, in the presence of anti-VISTA, anti-PSGL-1 or combination of anti-VISTA+anti-PSGL-1 antibody and corresponding IgG controls antibodies.

**(F**-**G),** Relative activation levels of CD8+ T cell, assessed as the percentages of IFN-γ+ CD8+ (**F**) and GZMB+ CD8+ (**G**) T cells (n=3 biological replicates). Error Bars, mean ± SEM. *P*-value, two-tailed unpaired *t*-test.

**
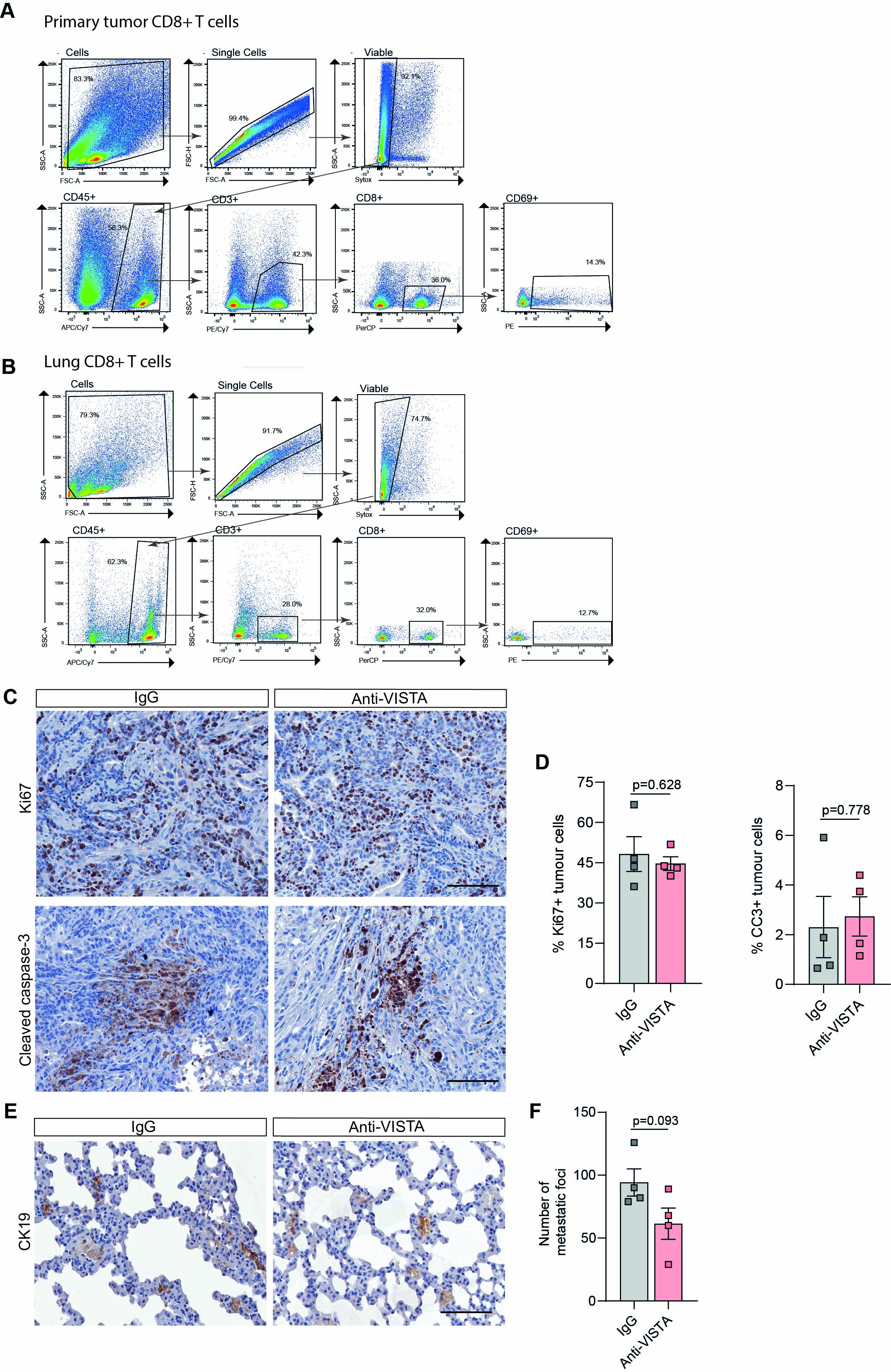
**

**Supplementary Figure-5 Analysis of T cell numbers and activation following VISTA blockade in primary tumour and metastatic lung tissues**

**(A-B)**, Gating strategy for flow cytometry analysis of CD8+CD69+ T cells in murine TNBC tumours (**A**) and lung tissues (**B**) form mice treated with IgG control antibody and anti-VISTA blocking antibody.

**(C)**, Representative IHC images of primary tumour tissue stained for Ki67 (proliferation marker) and cleaved caspase-3 (apoptosis marker) in IgG-treated and anti-VISTA-treated groups. Scale bar, 100μm.

**(D)**, Quantification of Ki67+ and cleaved caspase-3+ cells in primary tumours. Data are presented as the percentage of positively stained cells per tumour. (n = 4 mice/group). Error Bars, mean ± SEM. *P*-value, two-tailed unpaired *t*-test.

**(E)**, Representative IHC images of CK19 staining in lung tissue to assess metastatic foci in IgG-treated and anti-VISTA-treated mice. Scale bar, 50μm.

**(F)** Quantification of CK19+ metastatic foci in lung tissue from IgG-treated and anti-VISTA-treated groups. Data are presented as the number of metastatic foci per lung (n = 4 mice/group). Error Bars, mean ± SEM. *P*-value, two-tailed unpaired *t*-test.
