# Supplementary figures and images for: Macrophages suppress CD8 + T cell cytotoxic function in triple negative breast cancer via VISTA
Source: Br J Cancer. 2025 May 2;133(1):40–51. doi: 10.1038/s41416-025-03013-5 (PMC12238233; doi:10.1038/s41416-025-03013-5)

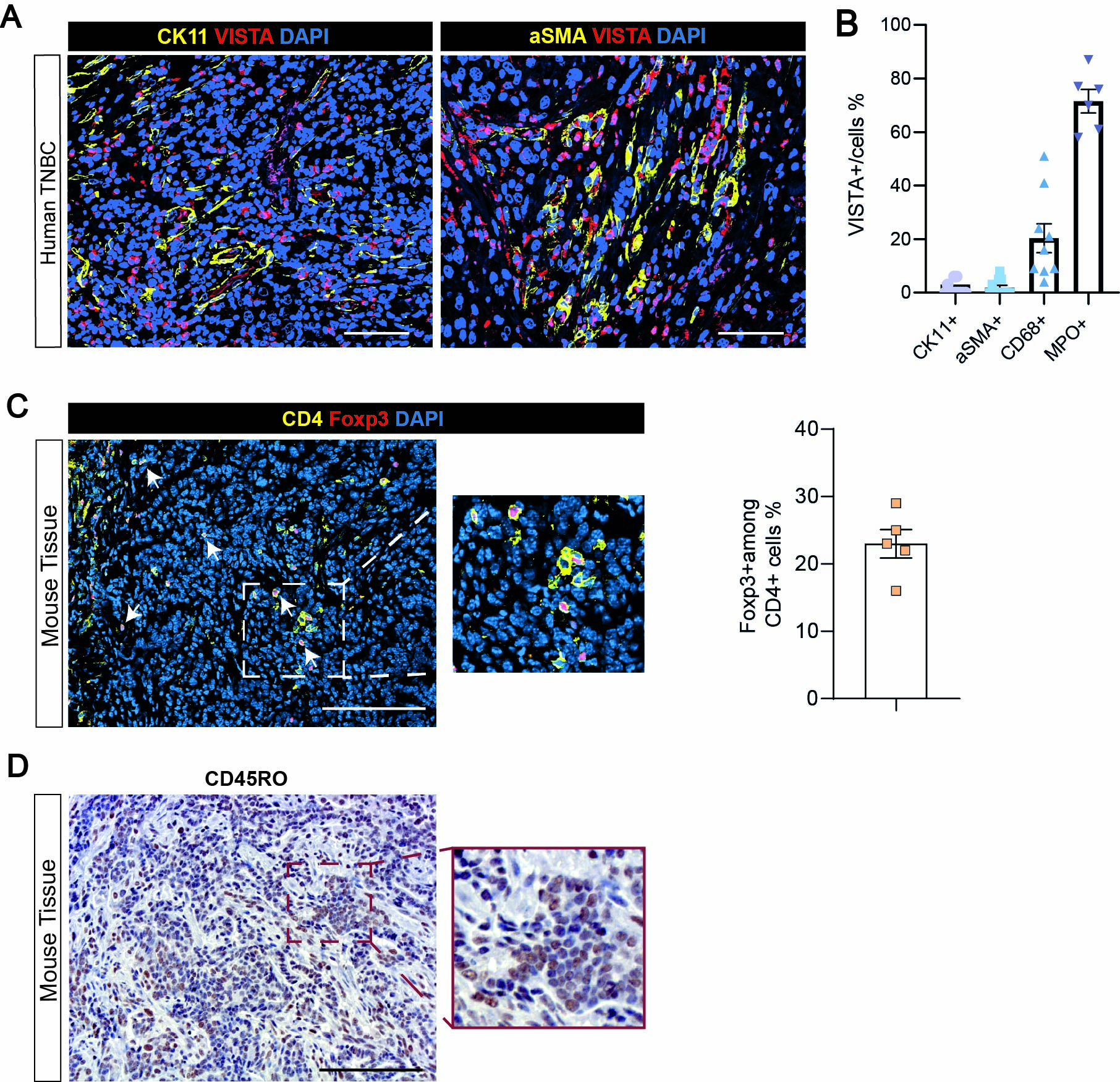

Supplement: Supplementary file 2 — Supplementary Figure 1 [file 41416_2025_3013_MOESM2_ESM.jpg]

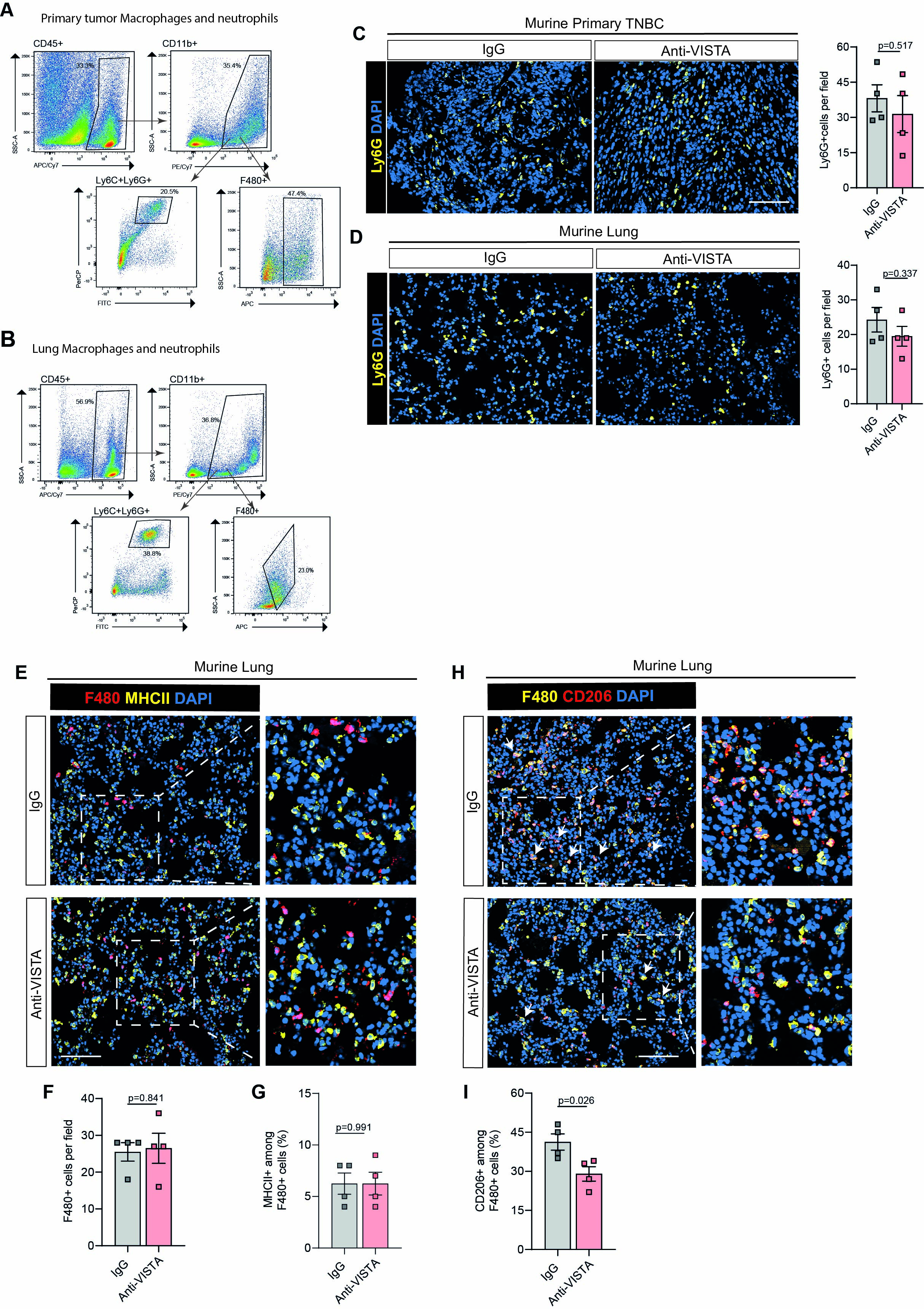

Supplement: Supplementary file 3 — Supplementary Figure 2 [file 41416_2025_3013_MOESM3_ESM.jpg]

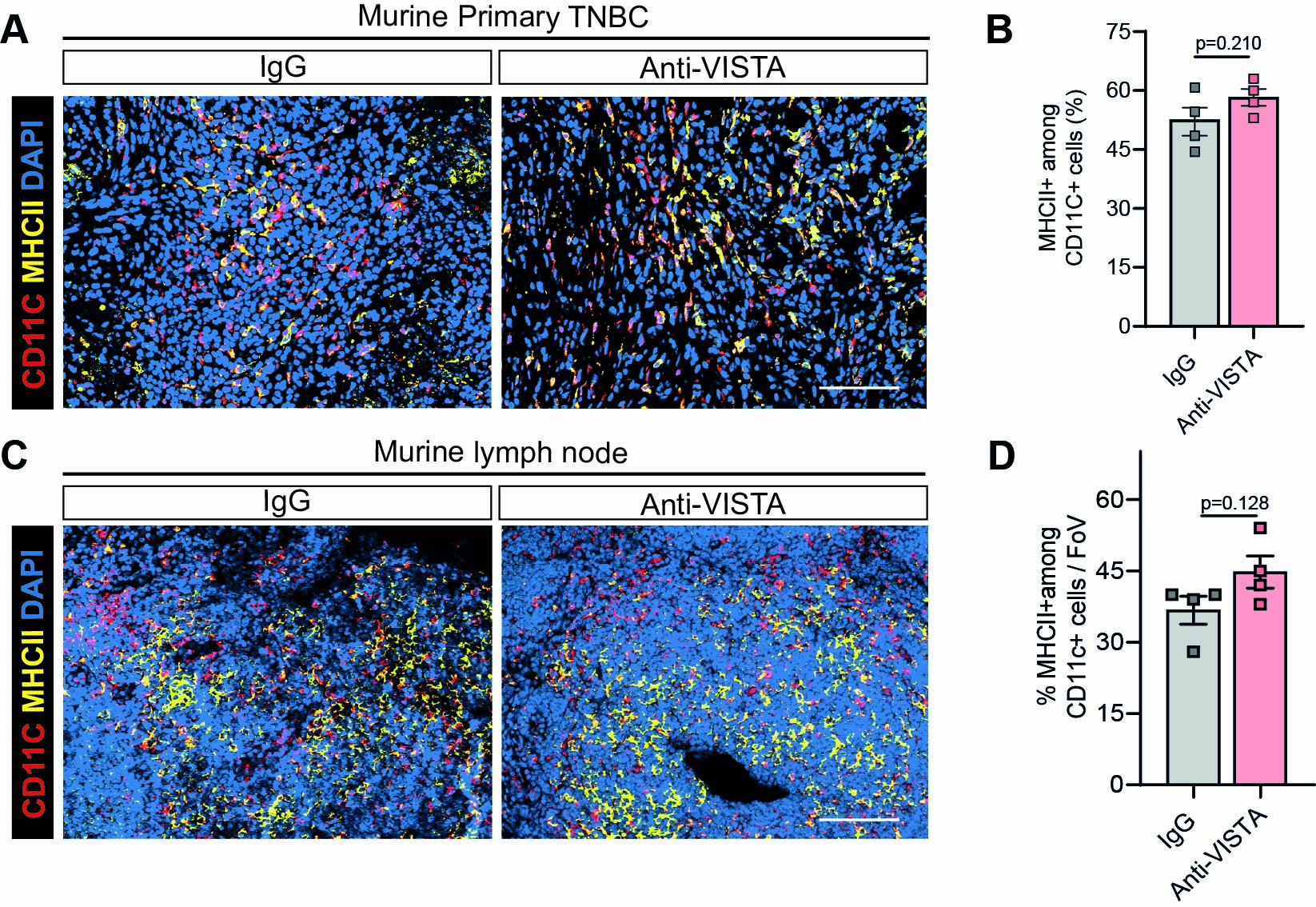

Supplement: Supplementary file 4 — Supplementary Figure 3 [file 41416_2025_3013_MOESM4_ESM.jpg]

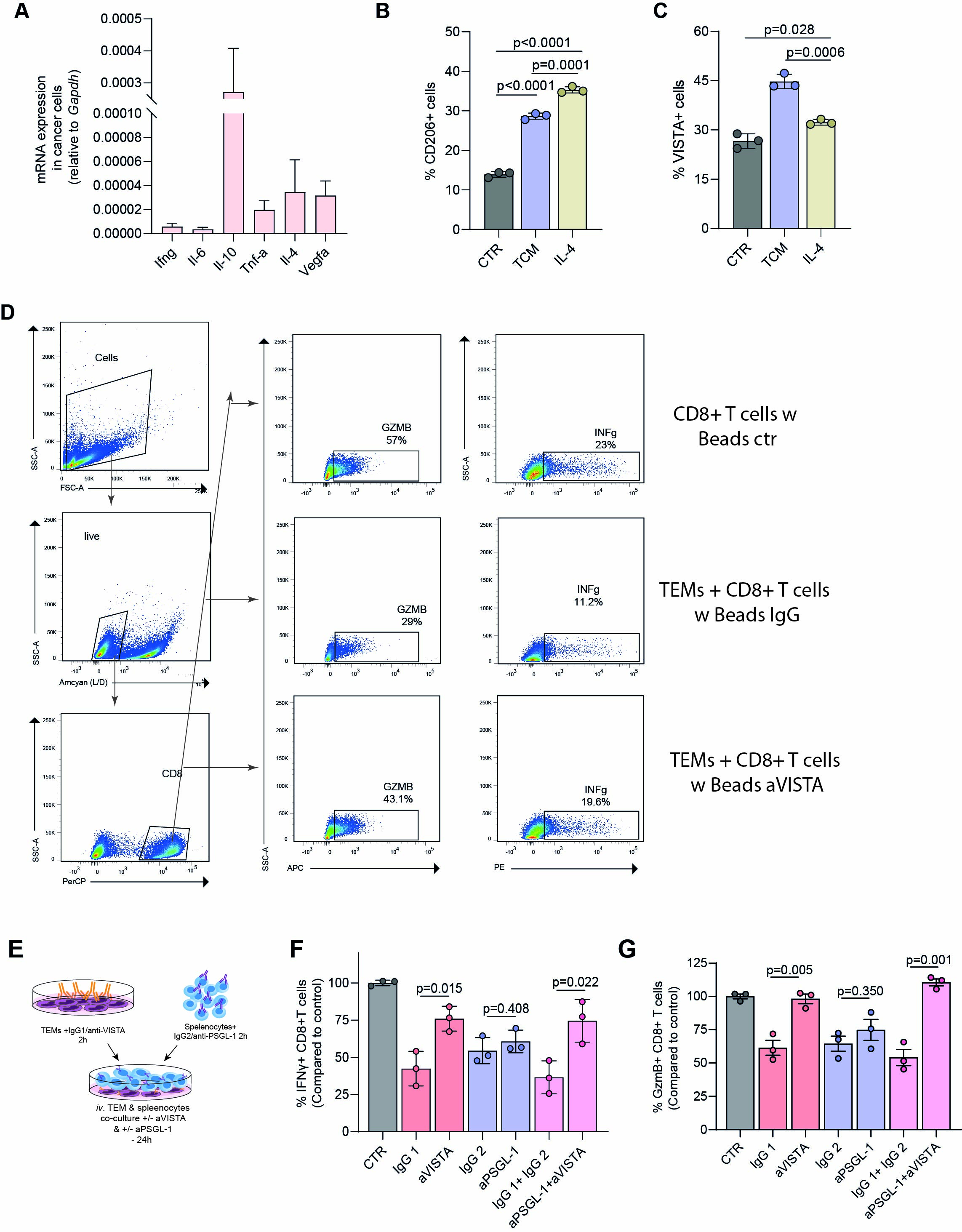

Supplement: Supplementary file 5 — Supplementary Figure 4 [file 41416_2025_3013_MOESM5_ESM.jpg]

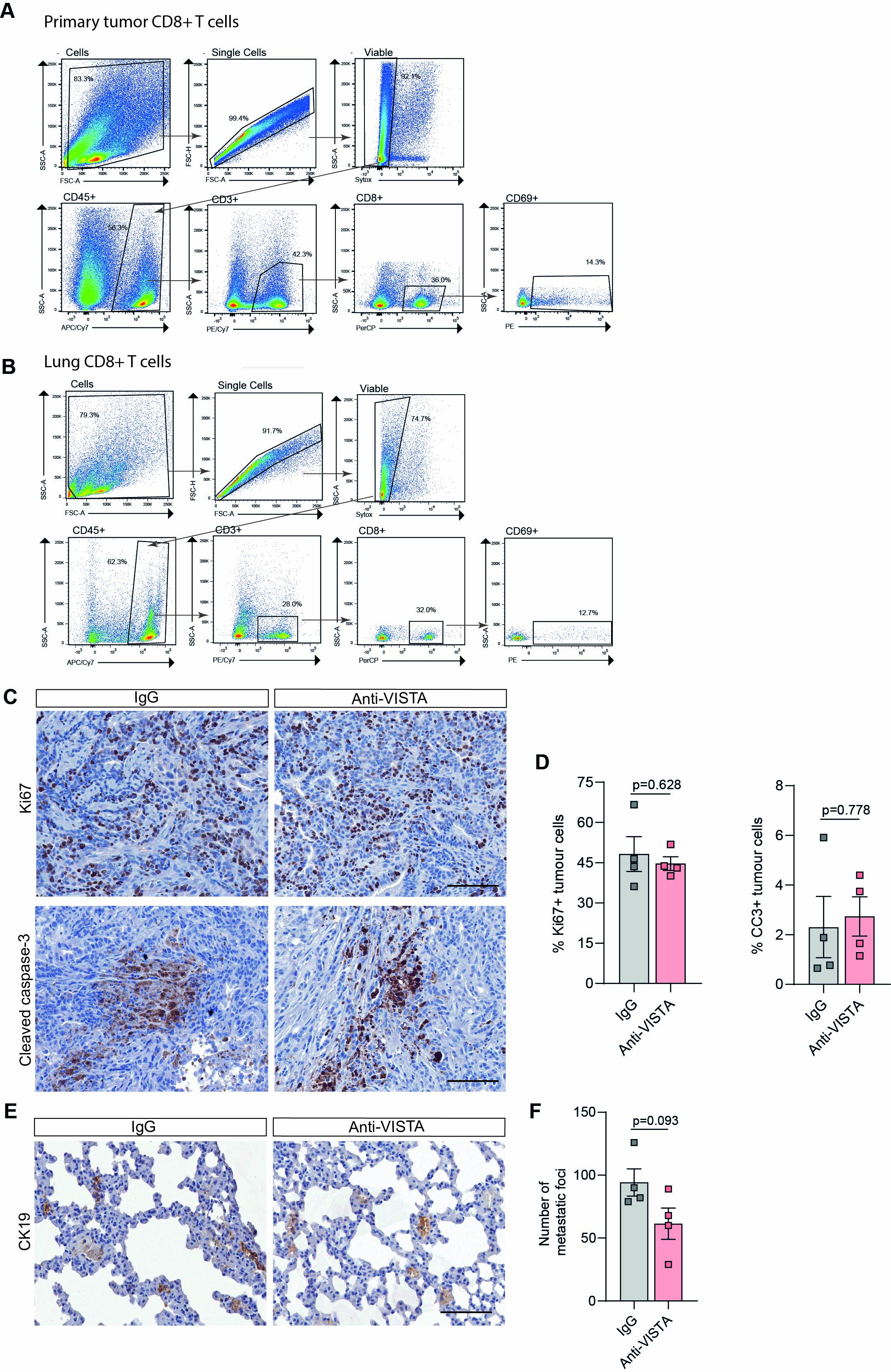

Supplement: Supplementary file 6 — Supplementary Figure 5 [file 41416_2025_3013_MOESM6_ESM.jpg]
