# Supplementary material for: Macrophages suppress CD8 + T cell cytotoxic function in triple negative breast cancer via VISTA
Source: Br J Cancer. 2025 May 2;133(1):40–51. doi: 10.1038/s41416-025-03013-5 (PMC12238233; doi:10.1038/s41416-025-03013-5)
Supplement: Supplementary file 7 — Supplementary tables [file 41416_2025_3013_MOESM7_ESM.docx]

**Supplementary Tables**

**Supplementary Table S1.** Antibodies used for Flow cytometry, immunofluorescence staining, and immunohistochemistry staining

**Supplementary Table S2.** Primers for quantitative real-time polymerase chain reaction

|  | |  | |
| --- | --- | --- | --- |
| **Murine qPCR primers** |  | |  |
| **Name** | **Sequence 5'-3'** | |  |
| Gapdh | Mm_Gapdh_3_SG QuantiTect Primer Assay (Qiagen) | |  |
| Arg1 | Mm_Arg1_1_SG QuantiTect Primer Assay (Qiagen) | |  |
| Ciita | Mm_Ciita_1_SG QuantiTect Primer Assay (Qiagen) | |  |
| Tnfa | Mm_Tnf_1_SG QuantiTect Primer Assay (Qiagen) | |  |
| Il-10 | Mm_ IL10_1_SG QuantiTect Primer Assay (Qiagen) | |  |
| Il-12b | Mm_Il12b_1_SG QuantiTect Primer Assay (Qiagen) | |  |
| Il-6 | Mm_Il6_1_SG QuantiTect Primer Assay | |  |
| Tgfb1 | Mm_Tgfb1_1_SG QuantiTect Primer Assay (Qiagen) | |  |
| Cxcl9 Forward | CAGCTCTGCCATGAAGTCCG | |  |
| Cxcl9 Reverse | TCCTTATCACTAGGGTTCCTCG | |  |
| Cxcl10 Forward | GTGTTGAGATCATTGCCACG | |  |
| Cxcl10 Reverse | TCTCTGCTGTCCATCCATCG | |  |
| Cd86 Forward | GATCAAGGACATGGGCTCGTA | |  |
| Cd86 Reverse | GGTTCACTGAAGTTGGCGAT | |  |
| Vsir Forward | CAACCTTCGGAGTCAGGACG | |  |
| Vsir Reverse | AGTCAGGGACTGGATCTAGGG | |  |
| IL-4 Forward | ACTGACCCCCAGGAGAACAC | |  |
| IL-4 Reverse | TCGTTGCGGGTCCTTTTC | |  |
| Vegf-a Forward | GAGATGAGCTTCCTACAGCACA | |  |
| Vegf-a Reverse | CACCAGGGTCTCGATTGGAT | |  |
| Infg Forward | AGGAACTGGCAAAAGGATGGTGA | |  |
| Infg Reverse | ACGCTTATGTTGTTGCTGATGG | |  |
